# Annotations

**Published:** 1899-12-16

**Authors:** 


### ANNOTATIONS.

### Charitable Institutions for Special Treatment.

AMONG the newer charities which are appealing to the public is one which goes by the name of the Tallerman Free Institutes, and apparently has for its object the establishment all over the country of institutions at which poor people may obtain the so-called "Tallerman treatment"; in other words, the use of the apparatus devised by Mr. Tallerman for the application of hot dry air to various parts of the body. Now we never like to say a word against charity of any kind, even though it is a virtue which does sometimes seem to cover a multitude if not of sins, at least of indiscretions, and we certainly do not wish to say anything against the treatment of rheumatism, stiff joints, and other conditions by the means of hot dry air, which we well know to be a most useful proceeding; but when it becomes a question whether it is wise for charitable people, or ethically correct for medical men, to support these proposed institutes, we confess that we have our doubts. There may possibly be room for a hospital for the special treatment of chronic rheumatism, and if charitable people choose to set up such an institution, fitted up with all the most modern forms of apparatus for the fulfilment of its purpose, we would wish it God speed. That, however, would be a very different affair from what is now proposed, which is to start a series of establishments for the carrying out of one particular line of treatment, by one particular sort of apparatus (one which in ten years' time may, for aught we know, be out of date), and for the advertisement of the "treatment" of one particular man, who, we believe, is not a medical man, but merely the contriver of the apparatus. If charitable people will combine to set up institutions at which the treatment of disease by hot dry air, by the aid of whatever form of apparatus is found to be the best, shall be placed at the disposal of the poor, all well and good. We do not think the plan would be ideal—first, because we do not approve of single-treatment institutions, and, secondly, because the whole thing could be done much better at the hospitals, if the money were given to them. All duplicate organisations are wasteful. Still, but for the waste, probably no great harm would be done. But, at any rate, let the patients have the best treatment possible; not the treatment of any Smith, Jones, or Robinson, or even Tallerman. Mr. Tallerman's apparatus is no doubt a good one. So are others. Mr. Tallerman himself, also, is no doubt an admirable man of business; but that is no reason why the charitable public should pay for his advertisements.

### Horse Ambulances for London.

A GOOD deal has been written on the subject of providing horse ambulances for London, but these writings have not always been prompted by knowledge. What has to be understood in considering the problem of how best to deal with the accidents and cases of sudden illness which occur so often in the London streets, is that London is so large a place, that the character of the traffic in different parts of the metropolis varies so greatly, and that the distance which has to be traversed before a hospital can be reached is so very different, according to the locality in which an accident occurs, that no general rule can be made applicable to all districts. One of our contemporaries, with sublime indifference to the facts of life and the exigencies of London traffic, holds that on the occurrence of an accident in the street the duty of the police should be merely to watch over the sufferer's safety, and to protect him from molestation till medical aid arrives, and that there should be a system by which medical aid and proper means of transport can be quickly summoned. Now, in regard to the out-districts, this is perfectly true. There can be no doubt that a properly organised system of horse ambulances is urgently required by those districts which are situated at a considerable distance from hospitals, and in which, from the lightness of the traffic, the police are not thick upon the ground. But in central London it is otherwise. There the police are practically within hail of each other, and it is impossible to put on one side the services of so admirably trained a body who, from the necessities of the case, must be the first on the spot, and whose duty it is to ensure the immediate removal of all obstructions to the traffic. It must not be forgotten that accidents of one kind and another are very numerous, and that under such a system as that suggested by our contemporary, namely, that of horse ambulances with house surgeons and dressers attached, standing ready to be called at the various hospitals, it would constantly happen that the ambulance would be engaged elsewhere at the moment when it was sent for, and that there would be delay in obtaining help. The proper solution of the difficulty is to be found in the provision of a complete system of horse ambulances, possibly with surgeons and dressers attached, for the out-districts where the distance to be traversed is likely to be considerable; and, for the busier districts, in the provision of a very much larger supply of wheeled hand ambulances than is at present available, and in the full utilisation of the police, who are always first on the spot. In regard to this we may also suggest that some official arrangment should be made for the payment of those who convey the patient to the hospital on these hand ambulances, a task which is not only laborious but far from pleasant when performed under the critical eye of a jeering London crowd.

### The One-Portal System.

THE proceedings of the General Medical Council took a somewhat unexpected turn when the motion made by Mr. Brown, in favour of the Royal Colleges in London combining with the Apothecaries' Society for the purpose of holding qualifying examinations conjointly, was passed by a considerable majority of those voting. If, indeed, some such arrangement could be arrived at, and if the London University could be persuaded to disembarrass itself of the work of examining for qualification, confining itself to higher education and to the granting of higher degrees, and admitting to its "finals" only those who were already on the register, we should have what would practically be a one-portal system, for London at any rate. But we doubt whether anything of the sort is likely to take place. Even if nothing else stood in the way it is to be feared that the high fees charged by the colleges for their qualifications would prevent the universities readily giving up their present right to grant a qualifying degree.

